# P177: Community-based learning: medical parasitology in pre-clinical year, Suranaree University of Technology, Thailand

**DOI:** 10.1186/2047-2994-2-S1-P177

**Published:** 2013-06-20

**Authors:** N Kaewpitoon, SJ Kaewpitoon, N Ueng-Arporn, P Wongkaewpothong, A Ngamnuan, T Olarnrachin, P Praphanpracha, K Rattanakereepun, N Namwichaisirikul, L Martrakul, V Vanapruek

**Affiliations:** 1 Pathology, Suranaree University of Technology, Nakhonratchasima, Thailand; 2 Suranaree University of Technology, Nakhonratchasima, Thailand

## Introduction

Medical Parasitology subject is one of basic medical science. We have integrated subject through community based learning.

## Objectives

Assess medical students’ attitudes toward practice in laboratory (TC) parasitological examination in the rural community (MPEC).

## Methods

Cross-sectional descriptive study was constructed among 46 medical students during April to July 2012. Attitudes were compared between practice in laboratory (TC) and mobile parasitological examination in the community (MPEC).

## Results

A total of 46 (*22 Males and 24 Females*) medical students. Most of students was highly satisfied with MPEC. Student’ skill, they could be identified parasites during community studied. A total of 85 stool sample was examined and found 7 samples were infected with hookworm (5 patients), *Strongyloides stercolaris* (1 patient) and *Taenia* sp. (1 patient), respectively.

## Conclusion

That modification in educational methods. MPEC experience in particular can favorably influence students' attitudes toward basic sciences.

## Disclosure of interest

None declared

